# Internet of Nano-Things (IoNT): A Comprehensive Review from Architecture to Security and Privacy Challenges

**DOI:** 10.3390/s23052807

**Published:** 2023-03-03

**Authors:** Abdullah Alabdulatif, Navod Neranjan Thilakarathne, Zaharaddeen Karami Lawal, Khairul Eahsun Fahim, Rufai Yusuf Zakari

**Affiliations:** 1 Department of Computer, College of Sciences and Arts in Al-Rass, Qassim University, Al-Rass 720223, Saudi Arabia; 2 Department of ICT, Faculty of Technology, University of Colombo, Colombo 00700, Sri Lanka; 3 Department of Computer Science, Federal University Dutse, Dutse 720102, Nigeria; 4 ZNRF University of Management Sciences, Dhaka 1212, Bangladesh; 5 Department of Computer Science, Skyline University Nigeria, Kano 700103, Nigeria

**Keywords:** Internet of Things (IoT), Internet of Nano-Things (IoNT), Internet of Everything (IoE), Information and Communication Technology (ICT), security, privacy, internet, cyber security, nanotechnology, wireless network, edge computing, fog computing

## Abstract

Throughout the course of human history, owing to innovations that shape the future of mankind, many technologies have been innovated and used towards making people’s lives easier. Such technologies have made us who we are today and are involved with every domain that is vital for human survival such as agriculture, healthcare, and transportation. The Internet of Things (IoT) is one such technology that revolutionizes almost every aspect of our lives, found early in the 21st century with the advancement of Internet and Information Communication (ICT) Technologies. As of now, the IoT is served in almost every domain, as we mentioned above, allowing the connectivity of digital objects around us to the Internet, thus allowing the remote monitoring, control, and execution of actions based on underlying conditions, making such objects smarter. Over time, the IoT has progressively evolved and paved the way towards the Internet of Nano-Things (IoNT) which is the use of nano-size miniature IoT devices. The IoNT is a relatively new technology that has lately begun to establish a name for itself, and many are not aware of it, even in academia or research. The use of the IoT always comes at a cost, owing to the connectivity to the Internet and the inherently vulnerable nature of IoT, wherein it paves the way for hackers to compromise security and privacy. This is also applicable to the IoNT, which is the advanced and miniature version of IoT, and brings disastrous consequences if such security and privacy violations were to occur as no one can notice such issues pertaining to the IoNT, due to their miniaturized nature and novelty in the field. The lack of research in the IoNT domain has motivated us to synthesize this research, highlighting architectural elements in the IoNT ecosystem and security and privacy challenges pertaining to the IoNT. In this regard, in the study, we provide a comprehensive overview of the IoNT ecosystem and security and privacy pertaining to the IoNT as a reference to future research.

## 1. Introduction

The growth and rapid demand for Information and Communication Technologies (ICT) early in the 21st century has influenced many domains such as manufacturing, transportation, communication, healthcare, and agriculture to reach their fullest potential and has led to the proliferation of many advanced sets of technologies such as the Internet of Things (IoT), Artificial Intelligence (AI), cloud computing, and so on [1]. Among such technologies, the IoT is recognized as a key technology that interconnects every digital device that prevails in the world, allowing them to communicate through machine-to-machine communication [2]. On the whole, as of now, the emergence of the IoT has profoundly altered the day-to-day functionality of everyone’s lives.

Progressively over the years, the IoT has transformed many domains such as healthcare and agriculture, allowing relevant stakeholders to connect every digital device that prevails in those domains to the Internet, facilitating fast information dissemination and integration with technologies such as AI to provide real-time analytics and real-time management facilities, making such domains smarter and ubiquitous [3,4,5]. Overall, the IoT represents sensors, actuators, smart mobile devices, and every prevailing digital device that can be integrated with the Internet that produces and exchanges a great deal of data [1,5,6]. With the aid of technologies such as AI, these generated data can be analyzed; to this end, technologies including cloud and fog computing offer convenient storage solutions for storing this IoT data, also facilitating further processing. With the help of the IoT, we can turn the objects around us into interconnected components of a vast and powerful ubiquitous network that operates on the free flow of data. Because of this, communication and the sharing of information throughout the globe have become much more seamless. Without a doubt, the IoT has revolutionized the way in which people use the Internet and device-to-device communication, that is, how devices, sensors, and objects interact with one another and exchange data, and it has also paved the way for the IoT on the nanoscale which is also referred to as the Internet of Nano-Things or IoNT [1,2,3]. Altogether, the IoT and the IoNT can be merged into a single term, the Internet of Everything (IoE) [3,4,5].

As of right now, the IoT is well past its adolescence and is prepared to take the globe by storm. On the other hand, the IoNT is a relatively new technology that has recently begun to make a splash. The IoNT is similar to the IoT but on a nanoscale [7,8,9,10]. In simpler terms, the IoNT refers to the interconnection of nano-sized devices within already existing networks [1,2,3]. The IoNT consists of networks of interconnected nanoscale devices, such as nanorobots, nanosensors, and nanomachines, that can communicate and exchange data with each other and with larger systems [10,11,12,13]. Overall, it is evident that these devices have the potential to revolutionize a wide range of industries such as agriculture and healthcare [4,5,6,14,15,16,17,18,19,20].

The IoNT is similar to the IoT in many aspects, with the primary distinction being that the devices that are interconnected within the IoNT are miniaturized and small enough to be termed nanoscale [1,2,5,6,7,8]. The range of dimensions for nanoscale devices is between 0.1 and 100 nanometers, with a nanometer being equal to one billionth of a meter [1,2]. The vast majority of technologies that are currently in use are examples of very small devices that can be miniaturized to fit within very small volumes. Some examples of these technologies include sensors found in automobiles and homes that report environmental conditions, as well as accelerometers and gyroscopes found in smart mobile devices that help in using navigation or location services [5,6,7]. These technological synergies fuel innovation in every conceivable field, from automobiles to healthcare and even our homes [12,13,14,15].

The applications of nanotechnologies that are included in an IoT system are highly varied depending on the context [16,17,18,19,20]. For example, smart manufacturing factories employ IoNT devices to monitor the environmental humidity, temperature, water temperature, water quality, and air pollutants [3]. On the other hand, modern automobiles equipped with similarly small sensors can make accurate predictions about proximity, environmental conditions, and position information, which helps to assure the safety and efficacy of vehicle assistance systems [3,4,5,6]. Further, in a typical smart city, in-place IoNT solutions are responsible for monitoring concentrations of toxic gases or particulates [4,5]. Devices would be planted at various locations throughout the city to monitor pollution levels in order to maintain the residents’ health and safety [20,21,22,23].

Accordingly, it is evident that the IoNT is being highly incorporated into most applications involved with our lives such as phones, household appliances, vehicles, and large-scale infrastructure systems [16,17,18,19,20]. The important fact is that not many have noticed that they are using these IoNT applications, owing to their miniaturized nature. These devices have their control and monitoring procedures digitized and connected to the Internet, which raises many security and privacy issues and is becoming a huge risk as of now [4,9,17]. As mentioned previously, owing to their miniaturized nature, many may have not noticed such security and privacy violations, if they have occurred, for a prolonged period of time. In such cases, the critical data can be easily breached by hackers as it is available via the Internet gateway of IoNT [7,8,9,10]. This may lead to damage to victims including theft, spying, and manipulation of their data [4,7,8,9,10,17]. Therefore, new security and privacy techniques are required to protect sensitive data collected by IoT nanosensors [4,8,9,10].

One of the main security risks of the IoNT is the potential for hackers to gain access to sensitive data transmitted by these devices. As these devices are often small and distributed in nature, it may be difficult to detect if they have been compromised [9,10]. In addition, the data transmitted by these devices may be sensitive in nature, such as medical data or financial information [4]. If these data were to fall into the wrong hands, it could lead to serious consequences for the individuals involved. Further, another risk associated with the IoNT is the potential for denial-of-service (DoS) attacks. These attacks occur when a malicious actor floods a device or network with traffic, rendering it unable to function properly [4,10]. As the IoNT consists of many small, interconnected devices, a DoS attack on one device could potentially affect the entire network [17]. This could have serious effects, particularly in cases where the IoNT is being used for critical infrastructure or emergency services. In addition to these security risks, the IoNT also raises privacy concerns. The proliferation of these devices means that individuals may be constantly monitored and tracked, potentially allowing companies or governments to gather vast amounts of personal data [4,9,10,11,12,17]. This data could be used for targeted advertising or could even be sold to third parties without the individual’s knowledge or consent which would tarnish one’s privacy.

Owing to the novelty in the field and as less research has been conducted in terms of the security and privacy of the IoNT, the eventual consequences of security and privacy breaches would lead to more disastrous consequences. Such consequences may include unavailability of services due to downtime of networks and servers, malfunction of IoNT solutions, tarnishing of the image of individuals and organizations due to privacy violations, and eventually, the loss of lives. Thus, motivated by the synthesis of the latest knowledge pertaining to the IoNT ecosystem and security and privacy challenges of the IoNT, in this study, we provide a comprehensive review of the IoNT ecosystem, including fundamentals and security and privacy issues, also highlighting the countermeasures. In this regard, the key contributions of the study are as follows:

1. Provides a comprehensive overview of the current status of the IoNT, applications of the IoNT, and benefits of the IoNT;
2. Provides a brief overview of the architecture of the IoNT, as fundamental vulnerabilities that exist in the architecture may lead to security and privacy challenges;
3. Provides a comprehensive overview of security and privacy issues pertaining to the IoNT including countermeasures;
4. Provides a summary of recent work pertaining to the subject, to differentiate our work from theirs.

The remainder of the study is organized in the following manner: In the second section, a brief overview of the IoNT, the architecture of the IoNT, applications of the IoNT, and the benefits of the IoNT are presented. In the next section, the current status of IoNT research is presented, summarizing the latest research work that has been performed. The next section focuses on the security and privacy of the IoNT. In the fourth section, we highlight the countermeasures for such security and privacy issues. Afterward, the future directions pertaining to the security and privacy of the IoNT are highlighted in Section 5. Finally, the study is concluded with the conclusions we derived through our work.

## 2. What Is IoNT?

In December 1959, Richard Feynman first presented the idea of nanotechnology [1]. Since then, nanotechnology has offered improved and effective solutions for various industries such as healthcare, agriculture, and so on [2]. In simple terms, nanotechnology is the study and use of microscopic objects measured at the atomic and molecular levels by combining various fields, including engineering, medicine, biology, physics, and chemistry; it has the potential to develop a variety of innovative technological materials and devices for the benefit of humanity [2,5,6]. Nanotechnology makes the development of devices with sizes between one and two hundred nanometers possible [3], leading to the evolution of microscopic components known as nanomachines. These are ordered collections of molecules that perform specific functions such as sensing or actuating [15,16,17,18,19]. This paved the way for a novel standard based on the IoT called “Internet of Nano Things (IoNT)”, which has been developed as a result of the connectivity of these nanosensors and nanodevices with exiting traditional communication networks within the Internet [2,4,5,19,20,21,22].

The concept of the IoNT was developed by Ian F. Akyildiz and Josep Miguel Jornet from the Georgia Institute of Technology in 2010 [10,12]. They describe the Internet of Nano-Things as “The interconnection of nanoscale devices with existing communication networks and, ultimately, the Internet defines a new networking paradigm that is further referred to as the Internet of Nano-Things” [2,12]. The IoNT is envisioned as being composed of nanoscale networks of physical items that are able to exchange information with one another via the use of nano-communication protocols; nanotechnology is seen as the fundamental pillar of the IoNT [1]. The IoNT paradigm encompasses many nanosensors to produce more accurate and reliable information about a specific object to improve our conceptual understanding of object behavior. It has the potential to significantly enhance the capabilities of the IoT, leading to a new generation of smaller, more efficient, and more capable devices [6]. One of the major advantages of the IoNT is that nanoscale devices are much smaller and more efficient than their macroscale counterparts [7]. This makes them ideal for use in applications where space is limited or where precise control is needed. For example, nanodevices could be used in with medical implants or wearable technology to monitor a person’s health [2,8,9,10]. On the other hand, when it comes to environmental monitoring, IoNT could be used to monitor air and water quality, providing real-time data on levels of pollutants and other contaminants [10]. This could help governments and businesses to identify and address environmental issues and could also help individuals to make more informed decisions about their health and well-being.

In general, the IoNT infrastructure can be deployed by combining nanodevices with the IoT, sensor networks, cloud computing, and big data analytics [1,4]. The IoNT has spawned numerous new domains, including the Internet of Bio-Nano-Things (IoBNT) and the Internet of Multimedia Nano-Things (IoMNT), which can aid in the development and integration of sophisticated technologies in the healthcare and media and entertainment industries [4,17,22,23,24,25]. The IoMNT architecture includes nano-cameras and photodetectors, which are constructed on a nanoscale and are mostly employed in telecommunications. The development of nanomachines with communication capabilities and the integration of nanomachines with micro- and macro-devices are factors driving the expansion of the worldwide IoNT market [23,26,27,28,29,30]. In reality, nanomachines are miniature devices that provide a variety of uses, including medicine delivery, food quality testing, and environmental monitoring [4,17].

According to recent market research [23], the size of the worldwide IoNT market is projected to reach USD 46.09 billion in 2028 and will have a revenue CAGR of 22.1% throughout the projection period. The primary drivers of the revenue growth of the worldwide IoNT market are increased government funding for the progress of nanotechnology, the growing incidence of numerous dangerous illnesses, and rising investments from the private sector [1,4,23]. 

### 2.1. Architecture of IoNT

Having established some context for the IoNT domain, we now turn to a brief discussion of the components that go into making up the IoNT’s architecture. To facilitate communication and data sharing at the nanoscale, the architecture of the IoNT is more involved with a wide range of technologies and protocols and is more complicated than that of the IoT [12]. Key components of the IoNT architecture include:

- Nanosensors and actuators

These are the primary building blocks of the IoNT; they can detect and measure various physical, chemical, and biological parameters, as well as perform actions based on this information. Examples of nanosensors and actuators include nanorobots, nanosensors, and nanocatalysts [1,4,7];

- Communication protocols

To enable communication between nanoscale devices, the IoNT relies on a range of communication protocols such as wireless communication technologies (e.g., radio-frequency identification (RFID), Bluetooth, near field communication (N.F.C.)) and wired communication technologies (e.g., nanowires, nanotubes) [1,4,7]. These technologies allow nanoscale devices to transmit and receive data over short distances;

- Data processing and storage

To process and store the large amounts of data generated by the IoNT, specialized data processing and storage technologies are required [5,6,7,8]. These technologies may include nanoscale memory devices, such as nanocapacitor-based memory, and nanoscale computing devices, such as quantum computing devices or molecular computing devices [1,4];

- Network Infrastructure

The IoNT relies on a network infrastructure that includes routers, switches, and other networking devices to enable communication between nanoscale devices and other devices on the Internet [8,9,10]. This infrastructure may include both traditional networking technologies and specialized technologies designed for the nanoscale.

Having provided an overview of essential components in the IoNT architecture, next we looked into the architecture of a single nanomachine as the foundation for more complex IoNT solutions. The following components make up a nanomachine:

- Control unit

The control unit regulates all other nanomachine components and is responsible for acquiring environmental data [5,6,18];

- Communication unit

The communication unit is responsible for sending and accepting data at the nanoscale [5,6,18];

- Reproduction Unit

The reproduction unit creates each component of the nanomachine using external components and assembles them to produce the nanomachine [5,6,18].

**Figure 1:**
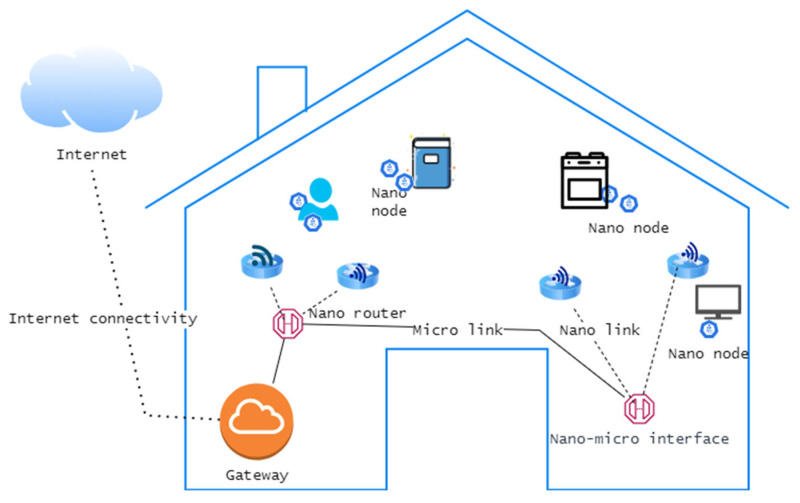
The IoNT network architecture for a smart home.

Overall, the architecture of the IoNT is complex and involves a wide range of technologies and protocols that enable communication and data exchange at the nanoscale. As the field of nanotechnology continues to advance, the capabilities of the IoNT are likely to expand, leading to a wide range of potential applications in many domains. Interconnecting nanomachines with current communication networks and, eventually, with the Internet, necessitates the creation of novel network architectures, which would depend on the application context. Having provided a brief overview of key components of IoNT architecture, next, we highlight the pervasive IoNT network architecture in the context of a smart home and an intrabody nanonetwork in healthcare. The IoNT architecture of a smart home is depicted in Figure 1.

**Figure 2:**
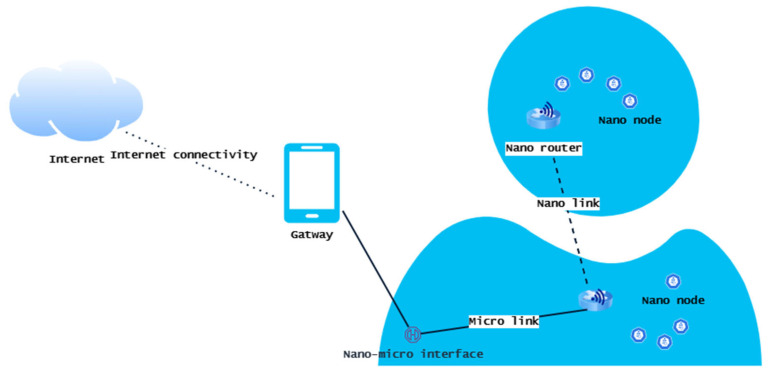
The IoNT network architecture of intrabody network in healthcare.

As shown in Figure 1, all components in the smart home environment have a nano-transceiver that allows them to be permanently linked to the Internet [1,4,7]. A tenant can easily keep track of the status of components in the home through this continuous Internet connectivity. On the other hand, as shown in Figure 2, in the context of an intrabody network, nanomachines are deployed inside the human body and remotely controlled at the macroscale over the Internet by relevant experts such as medical staff or healthcare service providers [1]. Owing to the portability of such IoNT-based healthcare solutions, the connection to the Internet is always provided through the data connection of mobile devices. In general, in the healthcare context, medical nanodevices can be deployed inside the human body or external environment and can also be used as wearable garments [11]. Depending on the deployed location, IoNT-enabled medical systems can be apportioned into three types: off-body systems (deployed anywhere based on the user preferences, such as in the home, vehicle, etc.), on-body systems (deployed on the person’s body), and intrabody systems (deployed inside the human body). Even though deployed location can vary, the architecture of a IoNT solution is always the same for all contexts [11].

**Figure 3:**
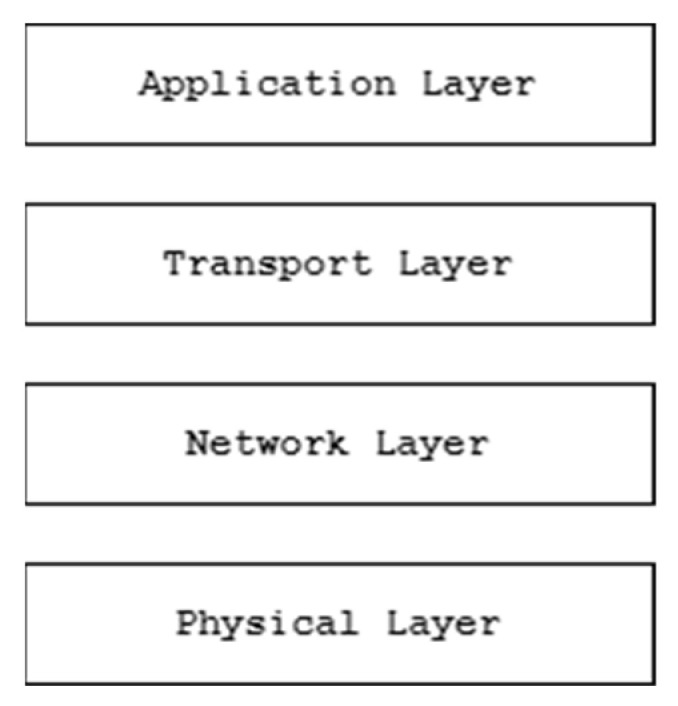
Layered network architecture of a generic IoNT solution.

Irrespective of the context wherein the IoNT solution is employed, the network architecture of a typical IoNT solution can be categorized into four layers, as shown in Figure 3.

According to the generic network architecture of a typical IoNT solution as depicted in Figure 3, the topmost layer comprises the application layer, which interacts mainly with the end user. The transport layer is responsible for the transmission of data back and forth between the application and the network layer. The network layer holds the responsibility for establishing nano-communication between nanodevices and the transport layer. The physical layer ensures guaranteed information delivery to the destination. Since the transmission range for nanodevices is limited, a thick arrangement of nanodevices is required at the physical layer [7,11]. As depicted in Figure 1 and Figure 2, to keep the connection alive and provide necessary services, the following described components in the network architecture hold greater responsibility:

- Nano-nodes

Nano-nodes are the smallest and most basic type of nanomachine; they are capable of performing very basic processing, storing a small amount of data in memory, and transmitting data over extremely short distances due to their low power and bandwidth [1,7]. Examples of nano-nodes include biological nanosensor nodes within the human body and nanomachines with communication capabilities integrated into a wide variety of objects in a smart home [1,17];

- Nano-routers

Nano-routers have comparatively larger computational resources than nano-nodes and are suitable for aggregating information coming from limited nanomachines [4]. Their functionality is very similar to that of the typical router in a computer network. In addition, nano-routers can also control the behavior of nano-nodes by exchanging very simple control commands (e.g., changing the on/off state). However, this increase in capabilities involves an increase in their size, and this makes their deployment more invasive;

- Nano–micro-interface devices

Interface devices aggregate information coming from nano-routers to convey it at the microscale and vice versa. On the other hand, these nano–micro interfaces act as hybrid devices and can communicate on the nanoscale using the aforementioned nano-communication techniques and use classical communication paradigms in conventional communication networks [1,4,5,6,7].

- Gateway

The gateway device enables the remote control of the entire IoNT system over the Internet. For example, as shown in Figure 2, in an intrabody network, a smart mobile device can forward the information it receives from a nano–micro interface to the healthcare provider over the Internet [1,4,5,6,7,15,16,17,18,19]. 

In addition, for IoNT networks to be completely operational, there has to be good integration and communication between nanodevices and macroscale components. Because of this, the construction of network topologies incorporating several communication paradigms, such as electromagnetic, acoustic, mechanical, and molecular communication, has seen exponential growth in recent years [30,31,32,33,34,35]. It is anticipated that nano-things would communicate with one another within these networks by sharing different sorts of information, such as synchronization signals, measured values of chemical and physical parameters, the outputs of logical operations, and instructions [2,3,4,5,36,37,38,39,40]. In this regard, molecular and nano-electromagnetic communication are the primary communication methods envisioned for use in IoNT networks. To send or receive information, molecular communication is established when specific molecules are released and then respond to other molecules [2,3,4,5]. On the other hand, the transmission and reception of electromagnetic radio-frequency waves in the terahertz (THz) band are required for nano-electromagnetic communication. However, there are challenges associated with using such communication paradigms, including coverage, compatibility, energy consumption, and the rate at which data may be sent [41,42,43,44,45]. Having provided a generic overview pertaining to the overall IoNT architecture, irrespective of the domains in which it is employed, in the next subsection, we provide a brief discussion on the applications of the IoNT.

### 2.2. Applications of IoNT

Based on the advent of technology, researchers have identified several potential uses of the IoNT spread across various fields that will have a significant future impact. At the moment that we are writing this, the IoNT has almost extended to almost all the domains that the IoT is served in. A brief overview of the applications of the IoNT, in each of the domains we identified, is provided as follows:

- Smart cities

Implementing a smart city allows interaction and communication with home appliances and the monitoring of various aspects pertaining to the city environment through deployed smart sensors, surveillance cameras, actuators, vehicles, and various other digital devices possessed by the citizens of the city, facilitating better management of the city. As of now, smart cities have already been established with the assistance of the IoT, wherein nanosensors are used to track and identify the location of litter found in high-absorption air regions and activate nanosensors to clear that area [1,17]. The IoNT has the ability to gather real-time data that may then be used to enhance public utilities, services, infrastructure, and much more in the context of a smart city. On the other hand, with the aid of IoNT solutions, the relevant parties in the city can gather real-time data to enhance the living standards of citizens living in the city [4,11];

- Oil and Gas

The IoNT offers convenient solutions to locate underground oil with high accuracy owing to the inherent characteristics of nanosensors [4,14]. The conventional technique for finding oil locations is less effective than this because it depends on a strong magnetic field and a receiver inside a particular system to send nanocomposites to the desired location. The ability of nanosensors to pass through rock holes effectively helps to find oil bound to the rocks. However, some seismic and cross-well imaging tools have a more significant impact within this domain, though their firmness level needs improvement [4]. On the other hand, nanosensors are able to collaborate and connect via molecular communication, wherein the collected data can be transmitted in real time by using the neighboring gateway. As a result, it is optional to use an exact magnetic source and receiver to plot the position of the oil [1,17];

- Agriculture

As of now, subject to various reasons such as volatile climate changes, the recent COVID-19 global pandemic, and pests and diseases, the world’s agricultural food production is challenged, even though many attempts have been made to overcome such challenges to provide food for the growing world population. This has already emphasized the need for smart farming/precision farming solutions, wherein the IoT acts as the main enabling technology of such solutions. In this regard, there are several ways that the IoNT is currently coupling with smart agriculture, for example, nanosensors are being used to monitor soil conditions, water usage, and crop growth, and to optimize resource management and reduce waste [1,2,3,4]

In general, nanosensors allow facilities to monitor crop health, soil quality, and moisture levels in real time. Further, with the aid of the IoNT, farmers are now able to collect extensive data to enable them to optimize their processes and, as a result, increase their yields [7]. Additionally, in terms of livestock management, nanosensors are used to track the location of farm animals such as cattle, control the feed given to farm animals, and keep a close eye on their health. Further, in terms of animal health, nanoscale biosensors are injected inside animals to monitor underlying conditions and these injected sensors offer unprecedented access to information about farm animals’ nutritional and medical needs [7,8,9,10].

- Healthcare

**Figure 4:**
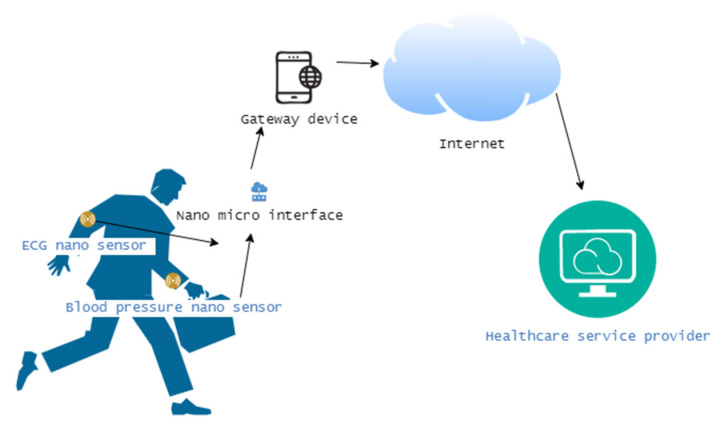
A typical IoNT application in healthcare.

Healthcare is a domain wherein IoNT solutions are highly used as of now, compared to all other domains, as we have noted through the reviewed state of the art. A Body Sensor Network (B.S.N.) is one such example. A B.S.N. employs various sensors implanted inside the human body to obtain pertinent insights and diagnostically valuable data that are otherwise outside the scope of traditional diagnostic methods [4,15,16,22,23,24,25,26]. Thanks to these nanoscale biosensors, surgeons can now access parts of the human body’s internal workings that were previously out of our reach. Further nanodevices could be used to monitor and track vital signs, diagnose and treat diseases, and deliver targeted therapies to specific areas of the body [26,27,28,29,30]. For example, nanosensors could be used to detect the early stages of disease and trigger an appropriate response, such as releasing a therapeutic agent [9,17]. On the other hand, the introduction of nanomedicine will lead to the development of better drugs and medical devices. It also offers different ways to improve medical diagnosis and treatment and helps in tissue and organ regeneration. Its applications also include early diagnosis of a wide range of diseases with high efficacy and personalization, which enhances patient treatment processes [22,23,24,25,26]. Moreover, the rising demand for faster and smaller portable diagnostic sensing systems is one of the major factors driving the deployment of the IoNT in healthcare. If the IoNT makes a breakthrough in the field of healthcare, doctors will be able to monitor patients in real time instead of having to measure manually as before. As shown in Figure 4, a typical healthcare IoNT solution comprising several nanosensors can communicate the gathered sensory data to an external device such as a smart mobile device or an internet gateway, enabling healthcare providers to keep a continuous eye on the patient’s underlying conditions [4,26,27,28,29,30].

- Environmental monitoring

In terms of environmental monitoring, nanosensors are used to monitor and track environmental conditions, such as air and water quality, soil conditions, and weather patterns [4,5,6,7]. This information could be used to improve resource management, protect the environment, and mitigate the effects of climate change [17]. In the context of a smart city, IoNT solutions are used to monitor real-time environmental conditions and generate alerts or responses according to underlying conditions [1];

- Manufacturing

The IoNT could be used to improve the efficiency and accuracy of manufacturing processes by enabling real-time monitoring and control of production lines. Nanodevices could be used to detect defects, track materials, and optimize production to reduce waste and increase efficiency [1,4];

- Transportation

The IoNT is used to improve the efficiency and safety of transportation systems. For example, nanosensors are used to monitor traffic patterns and optimize routes, or to detect and prevent accidents [2,4,17];

- Energy

The IoNT is also used to improve the efficiency and reliability of energy systems. In the context of the smart grid, nanodevices are used to monitor and optimize energy production and consumption, and to detect and prevent failures in the energy grid [4,10,11,12];

- Military

Over the years, military tactics have evolved towards higher damage utilizing fewer resources; in this respect, biological and chemical weapons have appeared as effective solutions. In the military, the IoNT can use nanosensors to detect the existence of chemical composites in concentrations of even only one molecule, paving the way towards identifying biological and chemical attacks [10,11,12,17]. Further, nanosensors can detect tiny flaws in bridges, civil constructions, cars, fabrics, and rockets. Nano-drones or Nano-Unmanned Aerial Vehicles (NUAV) are other aspects of the IoNT in the military that are used for surveillance activities and carrying explosives for military personnel [17].

### 2.3. Benefits of IoNT

Having provided an overview of the IoNT, its architecture, and relevant applications, it is evident that the IoNT is currently served in almost all the domains that the IoT is served in. In each of these domains, depending on the context, the IoNT performed better (as opposed to the IoT), owing to their miniaturized nature. Thus, to summarize, the potential benefits of the IoNT are outlined as follows:

- Improved accuracy and precision

As nanoscale devices are so small, they can be used to measure and monitor at a much higher level of accuracy as opposed to larger devices. This can be especially useful in applications where accuracy is critical, especially in medical diagnosis and treatment [1,4,15];

- Increased sensitivity

Nanodevices can be designed to be highly sensitive to a wide range of stimuli, such as changes in temperature, pressure, or chemical composition. This can enable them to detect and respond to changes in the environment that would be imperceptible to larger devices [2,4];

- Greater energy efficiency

As nanoscale devices are so small, they can operate on very low levels of power. This makes them ideal for applications wherein energy efficiency is important, such as in wearable devices and other portable electronics [7,12];

- Enhanced functionality

Nanodevices can be designed to perform a wide range of functions, such as sensing, computing, and communication. This makes them very versatile and allows them to be used in a wide range of applications [1,12];

- Miniaturization

One of the key advantages of nanotechnology is that it allows for the creation of extremely miniaturized devices. This could lead to the development of wearable or implantable IoT devices that are less obtrusive and more convenient to use [13,14];

- Improved durability

Nanoscale components are often more resistant to damage and wear than their larger counterparts. This could lead to longer-lasting IoT devices that require less maintenance and repair in the long run [1,15,16].

Overall, the IoNT has the potential to revolutionize many different industries and areas of society by enabling the creation of a vast network of interconnected devices and sensors that operate on a nanoscale. Thus, increasing awareness regarding the various benefits of the IoNT and the development of nanomachines will significantly boost the IoNT market in the long run. As of now, it has attracted significant interest from researchers and the industry. However, there are also challenges associated with the development and deployment of nanodevices, as the use of technology always comes at a cost. 

## 3. Current Status of the Research

The IoNT has the potential to find solutions to many of the challenging problems the world is currently facing. It functions similarly to how we connect devices on the IoT, the key difference being that it can combine nano-elements, which the IoT cannot. As a result, a groundbreaking revolution could be seen in domains in the near future wherein the IoT is served. Owing to the novelty in the field, only a few works of research have been conducted so far, as the technology is in its infancy age. In order to provide a status of the current research in the field, we provide a summary of the latest research work. In Table 1, we provide the reference, scope of the study, and research contributions made by the researchers.

According to the summary we have presented, it is evident that most of the recent work pertaining to the IoNT targets the healthcare domain, owing to the lifesaving benefits offered by such nanoscale IoNT solutions in terms of disease diagnosis and treatment for ailments. On the other hand, a great deal of research work focuses on overcoming communication challenges with regard to the IoNT. In contrast, only a few works of research have been conducted in terms of the security and privacy of the IoNT.

## 4. Security and Privacy of IoNT

The IoNT is sometimes referred to as a miniature form of the IoT that possesses a significant potential for incorporation into real-time applications. In spite of the fact that it offers unbounded advantages, the IoNT is plagued by a number of obstacles that need to be overcome before it can be considered an indispensable component of humans in the not-too-distant future and without any restrictions.

Among such challenges, difficulty in controlling and communicating with such small devices and concerns regarding the potential safety and environmental impacts of nanotechnology have become key research areas that researchers are currently working on [31,32,33,34]. On the other hand, when it comes to both the IoT and the IoNT, such solutions always have to be connected to the Internet, towards inter-communication and exchange of data. This provides an opportunity for hackers to gain access to and control nanodevices. If a hacker were able to gain control over a nanorobot, for example, they could potentially use it to gather sensitive information or disrupt the function of the device [4,6,46,47,48,49,50]. As the connection to the Internet is an essential component of the IoNT, even though various other challenges exist that hinder the growth of technology, the security and privacy challenges pertaining to the IoNT become the most alarming.

Most applications we use in our daily lives, including mobile devices, home appliances, sensors, vehicles, and massive infrastructure systems, incorporate the IoNT. These nanodevices are connected to the Internet and have digitalized control and monitoring processes, which poses various security and privacy concerns as the data collected by these devices may be sensitive or personal [1]. Because IoNT devices are miniaturized by nature, detecting or preventing the tampering of these devices may be difficult, making them more vulnerable to hacking or other cyber-attacks than the IoT [17,18]. As the devices cannot be seen by the naked eye, such security breaches may lead to more disastrous consequences, leaving no clues for the victims, unlike the cyber-attacks that target the IoT. On the other hand, when it comes to privacy, one major privacy concern with the IoNT is the potential for nanodevices to collect and transmit sensitive personal data. For example, nanosensors embedded in clothing or wearable devices could potentially collect data on an individual’s movements, health, and behavior [9,13]. This data could be used for targeted advertising or other purposes without the individual’s knowledge or consent.

Another major security threat to the IoNT is the potential for malicious actors to gain access and manipulate nanodevices [1,4,17]. Because these devices are often embedded in or integrated with larger systems, a breach of a single device could have far-reaching consequences, compromising the security and privacy of such larger systems. For example, a hacker could potentially gain access to a nanorobot used in medical procedures and alter its functionality by compromising the security of microscale network devices or endpoint devices through infected malware, leading to potentially fatal consequences [51,52,53,54]. Another threat is the potential for nanodevices to be used for surveillance and tracking. Because these devices are small and can be easily hidden, they could be used to monitor individuals without their knowledge or consent, which would violate one’s privacy. This could have serious implications for privacy and civil liberties, particularly if the devices are used by governments or other powerful entities. Owing to the fact that medical information should be protected from unauthorized access and that such access can have a significant impact on people’s lives due to the data’s sensitivity and confidentiality, the healthcare industry, which is one of the major domains that highly utilizes the IoNT, raises significant concerns about the privacy of medical data [31,32,33,34,35,36]. According to a previous publication [9], in a typical healthcare system, the attacker is able to attack private data, such as biological data gathered by either in-body or wearable sensors, disrupt medical applications in the form of denial-of-service attacks (this would lead to severe consequences as the legitimate instructions may not be able to reach to the destination/vital body organs on time), and modify the communication links at the nano-communication level or the gateway to the Body Area Network (BAN), posing a serious threat to the lives of patients as we have already mentioned [37,38,39,40,41,42,43,55,56,57,58,59,60]. To address these threats, it is important for organizations and individuals to implement strong security measures, such as encryption and authentication protocols, to protect against unauthorized access to nanodevices. It is also important for governments and industries to develop and enforce regulations and standards to ensure the secure and responsible development and deployment of these technologies [34,35,36].

The COVID-19 global pandemic, which started in December 2019, has influenced many domains across the world, owing to the deadly nature of the virus; it has killed nearly seven million people as of the date of publication. Owing to its contagious nature, the pandemic has changed the nature and working conditions of companies across the world. As employees continue to work remotely, companies need to automate their operations even further. IoNT developers are currently working on an implementation of available IoNT techniques, which will boost the growth of the IoNT market in the future. Day by day, the IoNT is becoming an essential part of our daily life and technology itself is reaching its maturity. Thus, it is essential to focus higher attention on the security and privacy of the IoNT, towards the growth of the technology. In this regard, the following subsection briefly discusses the security challenges the IoNT faces, and highlights the privacy issues of the IoNT.

### 4.1. Security of IoNT

The IoNT is subject to various security risks, similar to the IoT, owing to its inherent vulnerabilities. In terms of security, a potential attacker can compromise the security of the IoNT by concentrating on the following focal points, violating physical, network, and data security measures and exploiting software vulnerabilities:

1. Physical/hardware security

Because of their small size, IoNT devices are vulnerable to physical tampering and damage. This can make it difficult to secure them against unauthorized access or tampering [1,4];

2. Network security

IoNT devices often communicate over wireless networks, which can be vulnerable to hacking and other cyber-threats. Ensuring the security of these networks is essential to protect the data transmitted by IoNT devices [4,17];

3. Data privacy

The data collected by IoNT devices may be sensitive and protecting these data from unauthorized access is deemed essential. This includes both the data transmitted by the devices and the data stored on them. These data can be vulnerable to unauthorized access or misuse [9];

4. Software vulnerabilities

As is the case for any software, the software running on IoNT devices is subject to vulnerabilities that can be exploited by hackers. Ensuring that these devices are kept up to date with the latest security patches is important to prevent such attacks [4,16,20].

### 4.2. Privacy of IoNT

Nanodevices can pose privacy risks if they are not properly secured. The following describes some of the key loopholes that malicious users can use to violate the privacy of the IoNT:

1. Data collection

Nanodevices can collect and transmit a large amount of data about their surroundings and the people who come into contact with them [9]. These data could be used to track individuals’ movements and activities, potentially leading to privacy violations, if they are not adequately secured;

2. Data security

As with any connected device, nanodevices are vulnerable to hacking and data breaches [22]. If a nanodevice is hacked, the attacker could potentially access sensitive data collected by the device;

3. Location tracking and surveillance

Some nanodevices, such as those used for environmental monitoring, may be equipped with GPS or other location-tracking technology [23]. This could allow someone to track an individual’s movements in real time, potentially violating their privacy [4];

4. Lack of regulation

There is currently a lack of regulation around the use of IoNT devices, which can make it difficult to ensure that the privacy of such solutions is adequately protected [17].

Having provided a brief understanding of focal points that a typical attacker targets in a typical IoNT solution, in the next section, we highlight the security goals of the IoNT which every IoNT solution must satisfy to tighten the security and privacy of such solutions. These goals are as follows:

- Confidentiality

Refers to the practice of avoiding the exposure of sensitive information to third parties who are not authorized to access it. When it comes to the IoNT, the secrecy of data must be ensured both when the data is at rest and when it is being sent between various nanodevices [17,36,37,38,39,40,41]. On the other hand, communications that are sent back and forth between a sender and a receiver ought to be shielded from the view of any user who is malevolent or who has not verified themselves;

- Integrity

Refers to the maintenance of the consistency, accuracy, and reliability of data across its full existence; data must not be altered in transit, and precautions must be taken to prevent malicious users from altering data [36,37,38,39,40,41]. Integrity checks, such as those provided by digital signatures based on cryptographic hash functions, need to be made available not only on the microscale components of IoNT systems but also on the nanoscale communication devices as well as the gateways [61,62,63]. Further integrity checks can be carried out at each node engaged in the message exchange between the sender and the receiver, or they can be carried out solely at end-user systems;

- Availability

The availability of information to authorized persons should be consistent and straightforward. Consequently, in the context of the IoNT, a malicious user must never be able to interrupt or negatively impact the communication or quality of service offered by nanodevices or nanonetworks [35,36,37,38,39,40,41]. Thus, IoNT availability should be guaranteed for components operating at both the microscale and the nanoscale;

- Authenticity

Ensures that the source of message transmission is reliable and prevents the attacker from sending bogus messages [36,37,38,39].

In general, IoNT security goals have to be followed when developing, configuring, and deploying IoNT solutions to guarantee the security and privacy of the IoNT. Despite the fact that solutions are developed in such a way to achieve such goals, some challenges still exist which would provide potential ways for attackers to compromise vulnerabilities and breach the security and privacy of the IoNT. Thus, in this regard, in the next subsection, we provide a brief overview of such security and privacy challenges pertaining to IoNT.

### 4.3. Security and Privacy Challenges of IoNT

This section provides an overview of the security and privacy challenges that pose vulnerabilities and provide an avenue for malicious users to compromise the security and privacy of IoNT. In general, a vulnerability refers to the openness or susceptibility to attack or harm by malicious users. Malicious users may try to exploit vulnerabilities present in a nanodevice or a nanonetwork; thus, these vulnerabilities need to be handled by implementing necessary security methods. The challenges to the security and privacy of the IoNT are summarized as follows:

- Resource limitation

5. Due to their diminutive size, nanomachines are constituted of minimal resources, including limited processing power and memory, to perform only necessary tasks. This limits the adoption and integration of built-in security procedures with the IoNT, such as encryption, making such devices vulnerable to security and privacy issues [17,38,39,40,41]. Additionally, energy consumption is a significant concern since communication devices, such as nanotube-based radios, need substantial power to generate their payloads. Thus, when building IoNT solutions, balancing the tradeoff between available resources and inbuilt security procedures must be taken into account;

- Exposure to the Internet

Having continual connection to the Internet is an essential aspect of the IoNT for proper function and information sharing. This continuous connection to the Internet leads the IoNT into a vulnerable state as it paves the way for attackers to exploit available software and hardware vulnerabilities through the Internet [4,36]. Further, nanodevices are often composed of limited computation and memory capabilities, and without built-in security features, which makes them an easy target for cyber-attacks coming from the Internet [36,37,38,39,40,41];

- Key management

It is generally agreed upon that the distribution of security keys (e.g., public and private encryption keys) is the fundamental activity at the heart of virtually any key management system. Keys can be disseminated either by key pre-distribution before the deployment or before any data transmission takes place. Both of these methods are viable options. When a key has been compromised, it is necessary to have the ability to revoke access to that key. This problem is still one of the most difficult problems with sensor networks and IoNT systems [4,17,36,37,38,39,40,41]. Hence, it is vital to describe standard methods for creating shared keys and to explain how keys can be revoked when it becomes necessary to do so;

- Secure localization

In order to successfully carry out their functions, several applications that rely on nano-communication require the localization of nanomachines [4,17]. Absolute placement with nanoscale resolution is difficult to achieve due to differences in requirements between traditional sensor networks, which use different coordinate systems, and nanodevices; however, relative positioning may be more applicable. This is closely connected to security and ensures that only adjacent nanomachines are able to interact with one another, preventing interference from remote attackers;

- Lack of encryption

Owing to the miniaturized size and limited computing resources of IoNT devices, integration of inbuilt security mechanisms is not always possible as the encryption and decryption processes consume a lot of resources. The failure to encrypt sensitive data shared between nanodevices, whether on the nanodevice itself or nanonetworks, leads to many security challenges that become increasingly problematic, i.e., attackers are able to eavesdrop on the sensitive data that is being exchanged [36,37,38,39,40,41];

- Malware threats

In recent years, there is a growing trend of malware that targets IoNT solutions, especially microscale devices in nanonetworks. Continuous internet connection has made it easy for attackers to compromise IoNT solutions [36,37,38,39,40,41].

### 4.4. Security and Privacy Attacks

**Figure 5:**
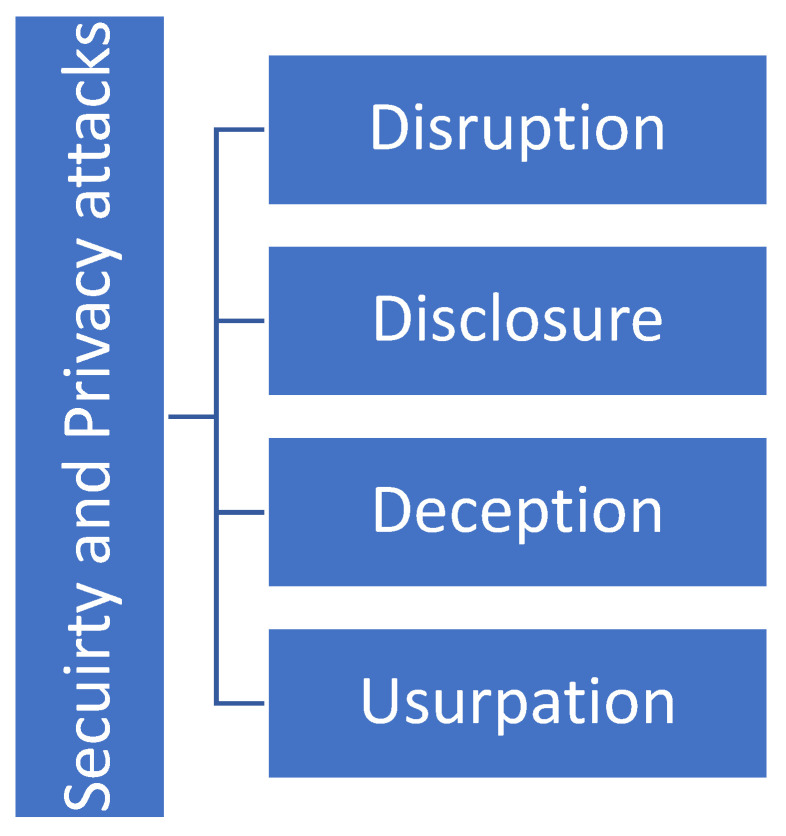
Categorization of security and privacy attacks.

Having discussed the security and privacy challenges pertaining to the IoNT, in this section, we provide a brief discussion on security and privacy attacks that target the IoNT ecosystem. In general, security and privacy attacks that target the IoNT ecosystem can be apportioned to four categories, as shown in Figure 5.

The security and privacy attack categories can be summarized as follows:

- Disruption

The availability of IoNT services and their dependability would both suffer as a result of disruption attacks [1,4];

- Disclosure

Malicious actors involved in disclosure attacks are able to gain access to sensitive information. When it comes to the IoNT, these kinds of attacks are difficult to launch when employing mechanical or molecular communication, whereas via electromagnetism and acoustics throw open the door for potential adversaries due to the comparatively broad region that is covered [9,17];

- Deception

Attackers’ ability to misrepresent or modify information is key in deception attacks [9]. Integrity checks are one method of putting a stop to these kinds of attacks;

- Usurpation

Beyond causing disruption to the system, unauthorized entities here have the ability to influence system services or entities [38,39,40]. Attackers can even gain complete control of the system in these types of attacks.

#### 4.4.1. Disruption Attacks

The following describes disruption attacks that target the IoNT:

- Flooding/denial-of-service (DoS) attack: It is possible to overwhelm the availability of potential IoNT nodes by delivering bogus messages [38]. The successful execution of a DoS attack by the attacker in this scenario has the potential to impede the availability of services provided by an IoNT network [40]. An aggressive variant of a denial-of-service attack known as a distributed denial of service, or DDOS, is characterized by the use of a greater number of compromised nodes to flood the system. This makes it more difficult to identify the origin of the attack. A wide variety of DDOS attacks can be launched by attackers by utilizing automated methods such as botnets [36,37,38,39,40].

#### 4.4.2. Disclosure Attacks

The following depicts the disclosure attacks that target the IoNT:

- Eavesdropping: The attacker spies on the communication between two IoNT nodes in the network [1,38,40];
- Jamming attack: The attacker disrupts communication between other nodes by creating noise in the communication channel [1,38,40];
- Sybil attack: Geographical routing protocols are disturbed by a malicious node claiming multiple identities [38,39,40];
- Man-in-the-Middle attack: This attack grants the attacker the opportunity to exploit a potential weakness, view and listen to data, and then, thereafter, replay and modify the data that is being conveyed in a covert manner [38,39,40].

#### 4.4.3. Deception Attacks

The following depicts deception attacks that target the IoNT:

- Spoofing/Replaying/Injection: Attackers try to become trustable by spoofing other nodes and broadcasting malicious information [1,7];
- Node capture: Nodes are captured in order to obtain cryptographic keys, and sensors can be reprogrammed to become malicious [36,37,38,39,40];
- Desynchronization attack: Sequence numbers of sent messages are manipulated by malicious nodes [36,37,38,39,40].

#### 4.4.4. Usurpation Attacks

The following depicts usurpation attacks that target the IoNT:

- Tampering of devices: The attacker can tamper with physical microscale IoNT devices and can manipulate their functionality or can fully or partially stop their functionality [37,38,39];
- Session hijacking: Session hijacking includes severe vulnerabilities in the session connection at the application interface, where an attacker can take control of the program session and application controls [40,41];
- Cross-Site Scripting (XSS): XSS attacks exploit applications by injecting malicious scripts into web pages to circumvent access control [40,41];
- Cross-Site Request Forgery (CSRF): In cross-site request forgery (CSRF) attacks, an attacker forces a currently authenticated end user to execute undesirable operations on a web application, resulting in catastrophic outcomes such as the disclosure of user credentials [37,38,39,40].

## 5. Countermeasures

To address the security and privacy issues associated with the IoNT, comprehensive security mechanisms must be implemented to protect against cyber-attacks and ensure that data collected by IoNT devices are handled securely and in accordance with applicable laws and regulations [40,41,62,63,64,65]. This may include encrypting data in transit and at rest, implementing strong authentication and access controls, and regularly updating software and hardware to address vulnerabilities. It is also critical to assess the privacy implications of IoNT devices and implement appropriate safeguards to protect personal data. There are a number of steps that can be taken to mitigate the security and privacy risks associated with the IoNT. One approach is to design devices with security in mind from the outset. This could involve using secure communication protocols and implementing robust authentication and access control approaches. It is also important to regularly update and patch devices to address any known vulnerabilities. Another approach is to implement strong privacy controls to ensure that individuals have control over their data and can choose what information is shared with others. This could involve providing clear and concise privacy policies and allowing individuals to opt out of data collection or sharing if they choose.

IoNT developers must implement strong encryption and authentication protocols to protect data transmission and prevent unauthorized access. It is also essential to consider the possibility of physical attacks and implement measures such as tamper-resistant hardware to prevent exploitation. In terms of privacy, IoNT devices need strict protocols for collecting and using personal data; this includes implementing opt-in consent for data collection and transparent privacy policies outlining the specific data being collected and how it will be used. Overall, the security of the IoNT depends on a combination of physical, network, and software security measures, as well as appropriate policies and procedures to ensure the protection of sensitive data. It will be necessary for manufacturers and developers of nanodevices to implement robust security measures and for governments and regulatory bodies to establish clear guidelines and standards for using and protecting personal data collected by these devices [59,60]. 

Further, owing to the small size of nanomachines and the fact that they operate on a nanoscale, they are more vulnerable to security and privacy breaches. In this discussion, we explore some of the countermeasures that can be implemented to ensure security and privacy on the nanoscale. One of the most important countermeasures for ensuring security within the IoNT is the use of secure communication protocols [4,17]. These protocols should be designed to encrypt all data transmitted between nanodevices and the larger IoT ecosystem. This will prevent unauthorized access to sensitive information and protect against attacks such as man-in-the-middle attacks. Another important countermeasure is the use of secure identification and authentication mechanisms for nanodevices. This can be achieved by implementing secure identification protocols such as public key infrastructure (PKI) and multi-factor authentication facilities [10,11,12,36,37,38]. These mechanisms ensure that only authorized devices can access the IoNT and prevent unauthorized access or manipulation of the nanodevices. In addition to secure communication and identification protocols, it is also important to implement security measures at the hardware level. This can include the use of tamper-proof enclosures for nanodevices, as well as the use of secure boot and firmware updates [36,37,38,39]. These measures protect against physical attacks and prevent unauthorized access to the device’s firmware. On the other hand, another important countermeasure to ensure privacy within the IoNT is the use of anonymization and pseudonymization techniques. These techniques can be used to protect the privacy of individuals by obscuring their personal information, such as their location or personal identification number. This can be achieved by using techniques such as k-anonymity or differential privacy [35,36,37,38,39,40]. Further, it is also important to implement strong data protection regulations for the IoNT [36,37,38]. These regulations should be designed to ensure that data collected and processed by nanodevices are used in a manner that is consistent with individuals’ privacy rights. This includes the right to be informed, the right to access, and the right to the erasure of data. Nonetheless, IoNT devices in healthcare are heavily regulated. As medical IoNT applications pose significant security risks, the European Union has created guidelines that precisely outline how medical devices must be handled and configured. As an example, Germany’s Medizinproduktegesetz (Medical Devices Act) refers to the national implementation of the European directives 90/385/EWG for active implantable medical devices [55,56,57,58,59,60]. Most regulations and directives classify medical devices into risk categories based on their duration of bodily contact, invasiveness, implantability, and impact on body function; IoNT systems and in-body networks are categorized as high-risk. 

In conclusion, the IoNT has the potential to revolutionize various industries, but its small size and the fact that it operates on a nanoscale make it vulnerable to security and privacy breaches. To ensure security and privacy within the IoNT, it is essential to implement countermeasures such as secure communication protocols, secure identification and authentication mechanisms, security measures at the hardware level, anonymization and pseudonymization techniques, and data protection regulations. By implementing these countermeasures, we can ensure the safe and secure operation of the IoNT while protecting individuals’ privacy rights. Further, there are several other countermeasures that can be taken to ensure security and privacy within the IoNT, mostly at the nanoscale level, which we have summarized in the following:

- Implement secure communication protocols such as HTTPS, SSL/TLS, and VPN to encrypt data transmitted over the network [36,37,38];
- Use robust authentication methods such as biometrics, multi-factor authentication, and digital certificates to prevent unauthorized access [1,2,3,4,9];
- Use firewalls, intrusion detection and prevention systems, and other security measures to protect against cyber-attacks [36,37];
- Regularly update software and firmware on devices to address known vulnerabilities [35,36,37];
- Use network segmentation to limit the scope of potential damage in case of a security breach [35,36,37,38,39];
- Use trusted hardware security modules to store sensitive data such as cryptographic keys [35,36,37];
- Use access controls and role-based access to limit access to sensitive data and functions [35,36,37];
- Conduct regular security audits and penetration testing to identify and address potential vulnerabilities [35,36,37];
- Implement data privacy measures such as data encryption and anonymization to protect personal data using cryptographic algorithms. However, in the context of the IoNT, it is noted that not all classical cryptographic mechanisms can be applied directly, including AES/RSA public or private cryptographic algorithms, owing to the reduced computational capabilities of nanosystems. According to references [4,17], and [29], lightweight biochemical cryptography is used in the context of the IoNT to encrypt sensitive data, and uses organic molecules such as DNA/RNA to encrypt data and protect the privacy and integrity of facts. However, private and public cryptographic algorithms can be used to safeguard the data on the macro-scale, such as AES, RSA, BLOWFISH, TWOFISH, etc. [1,4,10,11,12];
- To lessen the impact of malware including viruses, worms, and trojans on nanorobot functionality, anti-malware software could be used. However, anti-malware programs have to be optimized for use with such devices’ limited resources. Specifically, a nanodevice developed by Taiwanese researchers converts 0.32 W of near-infrared (NIR) light energy under the skin into electrical power [53,54];
- Have an incident response plan in place to quickly respond and mitigate any security breaches [36,37];
- Regularly train employees on best practices and policies regarding security to raise security awareness [36,37,38,39].

## 6. The Way Forward 

The IoNT is still a relatively new field, and many researchers are working towards making it a stable technology. The majority of the current research focuses on the potential uses of nanotechnologies in medical care and the design of hardware and software solutions for the IoNT. As we have noticed, concerns about security and privacy for the IoNT have not received much attention from academia or industry. In order to protect users’ security and privacy, it is crucial to concentrate efforts on coupling the current security systems used in traditional networks with nanonetworks or on implementing novel innovative solutions. Hence, in the following, we provide a brief discussion on anticipated future directions in terms of the security and privacy of the IoNT:

- Advancement in cryptography

With the development of quantum computing, classical cryptography may become less effective. Therefore, there is a need for new cryptographic techniques that can withstand quantum attacks. Post-quantum cryptography is a field that is actively being researched and developed to provide security for the IoNT [37,40,42,43,44,45];

- Blockchain-based solutions

The decentralization and immutability of blockchain technology can be applied to the IoNT to provide a secure and transparent way to manage the identities and interactions of nanodevices [36,37,38,39,40,44,45,46,57];

- Edge and Fog Computing

As the number of nanodevices connected to the IoNT increases, it will become increasingly challenging to manage and process the data generated by these devices. Edge and fog computing can be used to bring computation and storage closer to the devices, thus reducing the need to transmit sensitive data over the Internet [29,30,40];

- AI-based security

AI algorithms (machine learning and deep learning) can be used to detect and respond to security threats within the IoNT in real time [37,38]. This can include identifying and blocking malicious nanodevices, detecting and mitigating real-time denial-of-service and distributed denial-of-service attacks, and identifying and responding to suspicious network activity;

- Privacy-enhancing technologies

Privacy-enhancing technologies such as homomorphic encryption, multiparty computation, and differential privacy can be used to protect sensitive data while still allowing their use for data analysis and machine learning [37,38,46,47,48,49,50,58];

- Interdisciplinary research

Security and privacy within the IoNT require an interdisciplinary approach involving experts from various fields such as computer science, nanotechnology, physics, and biomedicine [44,45,46,47,51,52,56,59]. Further research in the IoNT will require collaboration between experts in these fields, and organizations and industries should provide continuous investments towards solving the privacy and security challenges in the domain [60].

In summary, the security and privacy of the IoNT are complex challenges that require a multi-faceted approach. Advancements in cryptography, blockchain-based solutions, edge and fog computing, machine learning-based security, privacy-enhancing technologies, and interdisciplinary research are some of the future directions that could be explored to ensure the safe and secure operation of the IoNT while protecting individuals’ privacy rights.

## 7. Conclusions

The rapid expansion of nanotechnology and its integration with the IoT has paved the way for the IoNT, which is exponentially growing with applications in smart cities, smart agriculture, the military, healthcare, and so on. Owing to its miniaturized nature and the significant benefits that can be expected compared to its counterpart, the IoT, the IoNT is becoming an essential part of our daily lives. Even though technology offers many benefits, the use of IoNT technology comes at a cost, owing to Internet connectivity, inherent vulnerabilities, and miniaturized nature, outweighing most of the benefits and posing significant security and privacy issues. Additionally, while developing such solutions, device manufacturers are not concerned about security and privacy; instead, they look into security once the devices are released to the market. This poses significant challenges once the devices are fully deployed, even leading to life-threatening situations in the field of healthcare, as healthcare is one such field that uses IoNT solutions extensively for disease diagnosis and treatments. Concerning these arguments, in the study, we have provided a review of security and privacy challenges pertaining to the IoNT. In the study, we have provided an overview of the IoNT and highlighted the current state of the research, the architecture of the IoNT, various applications and benefits of the IoNT, and the security and privacy of the IoNT. Regarding security and privacy, we have briefly discussed challenges pertaining to the IoNT that hinder the proper adoption of security and privacy. Further, we have categorized security and privacy attacks based on their attack vectors and also provided countermeasures and anticipated future research directions. Overall, we have underlined that security has to be an integral part of IoNT, whether it is on the microscale or nanoscale. Due to the miniaturized nature of the IoNT, rigid security- and privacy-preserving techniques have to be implemented on the microscale level to adequately protect the IoNT, even though security is weakened at the nanoscale.

## Figures and Tables

**Table 1:** Summary of the current status of the latest research.

Reference	Scope of the Study	Contributions
(I. Akyildiz and Jornet, 2010) [1]	IoNT communication	The researchers discuss the current state of electromagnetic communication among nanoscale devices, highlighting the major research challenges in terms of channel modeling, information encoding, and protocols for nanonetworks.
(Agarwal et al., 2017) [2]	Challenges pertaining to IoNT	The authors examine the key challenges of the IoNT, including security, privacy, and communication difficulties. The fundamental design of the IoNT has been described, and methods for overcoming the challenges have also been provided.
(Kulakowski et al., 2017) [3]	IoNT communication	The authors addressed how to route messages across nanonetworks and nano-communications using Forster resonance energy transfer (FRET), which was found to be a technology with very fast signal propagation speed. Based on the biological characteristics of specific molecules, they proposed five novel routing mechanisms, and one of these processes was empirically verified.
(Atlam et al., 2018) [4]	Challenges pertaining to IoNT	In this study, the authors examine the challenges and possibilities presented by the IoNT in a variety of application contexts. As security is regarded as one of the most important aspects of the IoNT, the authors present an in-depth analysis of its security objectives, attack vectors, and security challenges.
(Ali et al., 2016) [5]	IoNT in healthcare	Researchers highlighted the different IoNT network architectures and the architectural prerequisites for IoNT adoption in healthcare. In addition, they highlighted the key applications of the IoNT as well as the significant challenges associated with using this technology in healthcare.
(Ezz El-Din and Manjaiah, 2017) [6]	IoNT applications	The authors provided a thorough understanding of the Industrial Internet of Things (IIoT) and the Internet of Nano-Things (IoNT) by presenting a range of fundamental concepts, architectures, communication classifications, communication issues, applications, benefits, and future research directions.
(Kuscu and Akan, 2016) [7]	IoNT communication	The researchers explore theoretical and experimental concepts to give a thorough architectural perspective of nano-communication, focusing on its core principles and design needs. They provided an outline of networking advantages presented by the intrinsic capabilities of fluorophores under the idea of the Internet of Molecular Things.
(Hassan et al., 2022) [8]	IoNT communication	The researchers propose an energy-neutral event recognition framework using pulse position modulation in which the event information is transmitted by nanosensors that use the energy harvested from the event. In this framework, the authors use pulse position to identify transmitting nodes communicating with a single receiver.
(Dressler and Fischer, 2015) [9]	IoNT in healthcare	The authors investigated the difficulties and potential associated with linking Body Area Networks and other exterior gateways to in-body nanodevices. In this regard, a unique network architecture meeting the application needs was developed.
(Naser et al., 2021) [10]	IoNT architecture, applications, and challenges	The authors provided a systematic review of IoNT architecture, motives, applications, and challenges, reviewing work from 2015 to 2021.
(Pramanik et al., 2020) [11]	IoNT in healthcare	The researchers investigate the clinical and medical applications of various nanotechnology implementations and present an extensive review of nanotechnology, biosensors, nanobiosensors, and the IoNT. In addition, they provided multilayer taxonomies of nanotechnology, nanoparticles, biosensors, nanobiosensors, and nanozymes. Using several instances, the possible medicinal and clinical uses of these technologies are discussed in depth.
(Dabhi and Maheta, 2017) [14]	IoNT architecture, applications, and challenges	The researchers present an in-depth analysis of the IoNT, its architecture, benefits, and limitations, as well as its application areas, to aid in the advancement of research and to provide insight into how we might overcome the challenges and make use of the IoNT in many fields.
(Jarmakiewicz et al., 2016) [15]	IoNT in healthcare	The paper presents the IoNT operating in telemedicine in healthcare.
(Akyildiz et al., 2015) [16]	IoNT in biology	The researchers presented the notion of the Internet of Bio-Nano-Things, which combines synthetic biology and nanotechnology to create objects based on the control, reuse, modification, and reengineering of biological cells.
(Almazrouei et al., 2018) [18]	IoNT applications	In the study, the authors provided a brief review of the IoNT paradigm, highlighting communication approaches, architecture, and key applications.
(Miraz et al., 2018) [19]	IoT, IoE, and IoNT	The authors provided an extensive review on the IoT, IoE, and IoNT. In this regard, they highlighted the latest trends and advancements, and they further highlighted 21 significant current and future challenges.
(Maksimović, 2017) [20]	IoNT in healthcare	The authors discuss the roles of nanotechnology and the IoT in medicine and healthcare and seek to develop an understanding of nanoscale solutions and techniques by emphasizing their advantages and analyzing their possible hazards and concerns.
(Sharif et al., 2021) [21]	IoNT in healthcare	The researchers developed a fuzzy logic-based fault detection system designed for a medical IoNT architecture to detect the root cause and severity of faults that occurred in an in-body nanonetwork.
(Miraz et al., 2015) [22]	IoT, IoE, and IoNT	The authors provided an extensive review of the IoT, IoE, and IoNT.
(Senturk et al., 2022) [24]	IoNT Internet of Bio-Nano-Things (IoBNT), Internet of Biodegradable Things (IoBDT), and Internet of Ingestible Things (IoIT)	The authors provided an extensive survey on the IoNT, IoBNT, IoBDT, and IoIT, wherein they presented research challenges, potential applications, and open research areas.
(El-Fatyany et al., 2020) [25]	IoBNT security	Focusing on the security challenges pertaining to the IoBNT, the authors proposed a privacy scheme working on the top of the biocyber interface in the IoBNT paradigm.
(Lee et al., 2015) [26]	IoNT in healthcare	The authors proposed a wireless nanosensor network (WNSN) that would be useful for intrabody disease detection.
(I. F. Akyildiz and Jornet, 2010) [27]	IoNT applications	The authors provided a brief review of several IoNT application areas.
(Stelzner et al., 2016) [28]	IoNT in healthcare	The authors looked at the communication challenges pertaining to the IoNT and Body Area Networks (BANs).
(Al-Turjman, 2020) [29]	IoNT communication	Challenges pertaining to secure data communication in the context of the IoNT are presented in the form of a review wherein the authors discuss security issues and associated intelligence to be considered while managing such issues.
(Ali and Abu-Elkheir, 2015) [30]	IoNT in healthcare	The authors outline a vision of the ubiquitous healthcare ecosystem and its architectural requirements in order to incorporate nanonetworks.
(Verma et al., 2022) [31]	IoNT in healthcare	The authors provided a systematic literature survey of IoT- and IoNT-based wearable devices, and the role of 5G in the IoT for healthcare. Furthermore, they explained the usage of nano-integrated wearable devices in terms of healthcare, such as for curing, monitoring, and detecting diseases.
(Balasubramaniam and Kangasharju, 2013) [32]	IoNT applications and challenges	The authors provided a brief review of IoNT applications and challenges such as challenges pertaining to energy conservation and data analysis.
(Zafar et al., 2021) [33]	IoBNT security	With regard to overcoming challenges pertaining to IoBNT, the authors propose a framework that utilizes a Particle Swarm Optimization (PSO) algorithm to optimize Artificial Neural Networks (ANN) and to detect anomalous activities in IoBNT transmission. Their proposed ANN model shows an accuracy of 98.9% when detecting anomalies.
(Jornet and Akyildiz, 2012) [34]	IoMNT	The authors discuss the state of the art and major research challenges in the realization of the IoMNT. Fundamental research challenges and future research trends pertaining to the IoMNT are also highlighted.
(Nikhat Akhtar and Yusuf Perwej, 2020) [35]	IoNT architecture, applications, and challenges	The authors provide a brief review of the IoNT, its architecture and challenges, explain the role of IoNT in the global market, and discuss IoNT applications in various domains.

## Data Availability

Not applicable.
